# The Role of Fc-like Receptor 3 in the Pathophysiology of Rheumatoid Arthritis

**DOI:** 10.3390/genes16111318

**Published:** 2025-11-02

**Authors:** Paweł Dec, Paulina Plewa, Adam Kubisa, Andrzej Pawlik

**Affiliations:** Department of Physiology, Pomeranian Medical University, 70-111 Szczecin, Poland; pawel_dec@onet.pl (P.D.); paulina.plewa@op.pl (P.P.); adam.kubisa183@gmail.com (A.K.)

**Keywords:** Fc-like receptor 3, rheumatoid arthritis, pathophysiology

## Abstract

The pathogenesis of rheumatoid arthritis involves a complex interplay of genetic predisposition, environmental factors, and autoimmune mechanisms that lead to chronic inflammation of the synovial membrane. Fc-like receptor 3 (FcRL3) is a receptor encoded by the *FCRL3* gene, located on the long arm of chromosome 1 at 1q23.1. Polymorphisms in the promoter region of *FCRL3*, rather than elsewhere in the gene, primarily affect the level of protein expression, which is of clinical significance. Understanding the structure of FcRL3, particularly in the context of genetic variants, is therefore important for elucidating the pathogenesis of autoimmune diseases. Detailed knowledge of the molecular architecture of immune receptors such as FcRL3 is also essential for advancing our understanding of immune function and for guiding the development of targeted therapeutic strategies in autoimmune disease. In this article, we discuss the role of FcRL3 in the pathophysiology and potential therapy of rheumatoid arthritis.

## 1. Introduction

Rheumatoid arthritis (RA) is an inflammatory disease and is classified as a systemic autoimmune disease which, because of its systemic nature, shortens life expectancy and diminishes quality of life. It is estimated that RA affects approximately 0.5–1.0% of the global population [1]. The condition places a significant burden on healthcare systems—direct and indirect costs (treatment, hospitalisation, loss of productivity) amount to tens of thousands of dollars per patient per year in developed countries. Moreover, the disease is associated with an increased risk of cardiovascular disease, infection, and higher mortality compared with the general population [2,3]. Women are approximately three times more likely to develop RA than men [4]. Onset usually occurs in the fourth or fifth decade of life. A more detailed understanding of the disease mechanism can be gained by elucidating the molecular processes that drive the development of RA. The heritability of RA is estimated at approximately 60% [5]. The risk of RA developing in the second identical twin when one twin is affected is approximately 12–15%, highlighting the scale of the genetic component, although this is still lower than initially assumed. The rate of co-occurrence in fraternal twins is estimated at 4% [6]. Variations in *HLA-DRB1* alleles have been shown to account for roughly 30% of the genetic contribution to RA [7]. The presence of certain *HLA* alleles increases the risk of developing RA by about two- to three-fold [8].

In the case of other genes (i.e., those not belonging to the major histocompatibility complex) whose variations influence the risk of developing RA, particular attention should be given to polymorphisms in the *FCRL3* promoter region—specifically, single-nucleotide changes in this area. The 152 single-nucleotide polymorphisms (SNPs) identified here may account for up to 15% of inherited cases of RA [9]. Many of these variations predispose not only to RA, but also to systemic lupus erythematosus and autoimmune thyroid diseases [10,11]. FcRL3 is a receptor encoded by the *FCRL3* gene, located on the long arm of chromosome 1, in the 1q23.1 region. The fact that these polymorphisms occur within the promoter, rather than elsewhere in the gene, indicates that they primarily affect the level of protein expression, which is of clinical significance. It is suspected that in the future, *FCRL3* gene diagnostics may support assessment of disease course and inform therapeutic decisions. Another potential avenue may involve targeting the product of this gene, i.e., the FcRL3 protein.

The pathogenesis of RA involves a complex interplay of genetic predisposition, environmental factors, and autoimmune mechanisms, leading to chronic systematic inflammation, including the synovial membrane of the joints. The early stages of the disease are marked by a breakdown in immune tolerance and the emergence of autoreactive T and B cells, which produce autoantibodies, primarily anti-citrullinated protein antibodies (ACPAs) and rheumatoid factor (RF) [12,13]. Activation of CD4^+^ T cells (particularly Th1 and Th17 subtypes) results in the secretion of pro-inflammatory cytokines [tumour necrosis factor-α (TNF-α), interleukin (IL)-6, IL-17], which stimulate synovial fibroblast proliferation and osteoclastogenesis, ultimately causing cartilage and bone destruction [14,15]. Antigen-presenting cells (macrophages, dendritic cells) also play a key role, sustaining the inflammatory response through the expression of costimulatory molecules and the production of chemokines that recruit further effector cells into the joint [16]. Dysregulation of the balance between effector and regulatory mechanisms (e.g., a reduced number of functional Tregs) contributes to the chronicity of inflammation and disease progression [17].

With regard to RA, reduced *FCRL3* expression would lead to an increase in the number of Tregs, which could more effectively suppress the inflammatory response [9]. FcRL3 functions as an inhibitor of T cells [18]. High levels of FcRL3 observed on CD8^+^ and TCRγδ+ T cells are mainly associated with those subpopulations that exert a suppressive function [9].

Clinically, both in diagnosis and in monitoring disease progression, commonly used RA markers remain valuable. These include RF, present in approximately 70–80% of patients; ACPAs, notable for their high specificity; and the erythrocyte sedimentation rate and C-reactive protein level. Additional haematological changes may also be observed, such as moderate erythrocyte reduction (anaemia of chronic disease), thrombocytosis, mild leucocytosis, and increased plasma concentrations of alpha-1 and alpha-2 globulins [19]. Analysing the co-occurrence of these features with specific *FCRL3* gene variants may prove useful in phenotyping patients with RA.

## 2. Material and Methods

**Objective:** The aim of this article is to identify and analyze the current state of knowledge on the role of the FcRL3 receptor in the pathogenesis and potential treatment of rheumatoid arthritis, with particular emphasis on the influence of *FCRL3* gene polymorphisms on protein expression and autoimmune mechanisms.

**Data Sources:** Searches of PubMed and Scopus were made covering literature from 1998–2024.

**Keywords Used in Article Selection:** Fc-like receptor 3, *FCRL3* gene polymorphism, rheumatoid arthritis, immunoreceptors, Fc receptor-like proteins, synovial inflammation, autoimmune disease, molecular pathogenesis, targeted therapy.

**Article Selection Process for Review:** The selection process for this review was planned to ensure clarity. The literature search was conducted in two reputable databases: PubMed and Scopus. Publications published in English or Polish between 1998 and 2024 were included. Combinations of keywords such as “Fc-like receptor 3,” “*FCRL3* gene polymorphism,” “rheumatoid arthritis,” “immunoreceptors,” “Fc receptor-like proteins,” “synovial inflammation,” “autoimmune disease,” “molecular pathogenesis,” and “targeted therapy” were used to identify relevant publications. Boolean operators AND and OR were used to narrow or broaden the search results. Only original articles, systematic reviews, or meta-analyses that addressed the role of the FcRL3 receptor or *FCRL3* gene polymorphisms in the pathogenesis of rheumatoid arthritis were included. Specifically, studies describing molecular mechanisms, genetic relationships, receptor expression levels, and potential therapeutic options were included. However, editorial comments, conference abstracts, and publications not addressing FcRL3 in the context of autoimmunity were excluded from the review.

**Limitations in selection:** Despite the systematic approach to search and selection, the process was subject to several significant limitations. First, the review included only articles published in English, which may have resulted in the omission of valuable studies available in other languages. Furthermore, due to the exclusion of editorial comments and conference abstracts, it is possible that reports containing preliminary but potentially important observations were omitted. It is also worth noting that the review focused on a qualitative analysis of available data, without conducting a meta-analysis, which limits the ability to quantitatively assess the strength of the association between the presence of *FCRL3* gene polymorphisms and the risk of developing rheumatoid arthritis. Furthermore, some of the available studies differed in terms of methodology, study populations, and laboratory techniques used, which hindered direct comparison of results and could have impacted the consistency of the final conclusions.

**Study Design:** Publication selection was performed by two independent authors who first reviewed the titles and abstracts, and then the full texts of eligible articles. In case of discrepancies, decisions were made jointly or in conjunction with the other authors. Data from selected studies were collected using a form that included information on the year of publication, study design, population characteristics, genetic variants analyzed, methods for determining FcRL3 expression, and the most important results regarding pathogenesis and potential therapeutic options. However, no new statistical analyses or meta-analysis were performed as part of this study; the review is qualitative and descriptive in nature, focusing on synthesizing the current state of knowledge.

**Review period:** The literature review covers publications published between 1998 and 2024. This timeframe was selected to capture both the first scientific reports on the role of the FcRL3 receptor and the latest studies examining its importance in the pathogenesis of rheumatoid arthritis and its potential therapeutic applications. This timeframe allows for a comprehensive analysis of the development of knowledge in this research area over the past two decades.

## 3. Biology of FcRL3: Classification, Functions, and Structure

FcRLs, also historically referred to as Fc receptor homologue (FCRH) or immunoglobulin superfamily receptor translocation associated (IRTA), are a group of surface proteins structurally related to the classic Fc receptors for immunoglobulins (FcR). To date, eight members of this family have been identified in humans: FcRL1–FcRL6, FcRLA, and FcRLB. These proteins are characterised by distinct patterns of tissue expression and diverse immunological functions [20,21].

FcRL proteins possess extracellular domains resembling immunoglobulin (Ig)-like domains, a transmembrane region, and a cytoplasmic tail containing either activating motifs [immunoreceptor tyrosine-based activation motif (ITAM)] or inhibitory motifs [immunoreceptor tyrosine-based inhibitory motif (ITIM)], thereby enabling modulation of cell signalling [22,23]. FcRL3, encoded by a gene located on chromosome 1q21–23—a region associated with several autoimmune diseases—is of particular interest in the context of autoimmunity [24]. Genetic variants of *FCRL3*, especially the −169C/T polymorphism (rs7528684) within the promoter region, can influence protein expression and thus alter susceptibility to autoimmune diseases, including RA, systemic lupus erythematosus, and autoimmune thyroid disorders [25].

It is known that FcRLs regulate the functioning of many immune cells, with *FCRL3* expression in Treg cells being of particular significance [9]. FcRL3 is a membrane glycoprotein that transmits signals through its cytoplasmic domain. Its structure comprises a cytoplasmic tail, a transmembrane domain, and an extracellular region. Signal transduction is mediated by the ITAM and ITIM [20].

The *FCRL3* gene polymorphism mentioned earlier involves the promoter region, specifically the −169C/C variant. This change increases the likelihood of NFκB transcription factor binding, thereby enhancing promoter activity and upregulating *FCRL3* mRNA transcription. Elevated FcRL3 expression, in turn, contributes to abnormal immune activation and loss of tolerance to self-antigens. One key mechanism is the correlation between high FcRL3 expression and dysfunction, alongside increased expression of the programmed death receptor PD-1, which results in reduced proliferation of regulatory T cells in carriers of the *FCRL3* variant compared with controls [21]. Studies have further shown that increased FcRL3 expression in regulatory T cells, cytotoxic CD8^+^ T cells, and γδ T cells is associated with an elevated risk of RA manifestation [22].

FcRL3 is a type I glycoprotein embedded in the cell membrane, characterised by a typical structural pattern: an extracellular N-terminal domain, a single hydrophobic transmembrane helix anchoring the protein within the membrane, and an intracellular C-terminal domain facing the cytoplasm [23,24]. The full sequence of human FcRL3 comprises 734 amino acids [23].

At the beginning of the chain (residues 1–17) lies a signal peptide—a short amino acid fragment that directs the nascent FcRL3 protein to the endoplasmic reticulum during biosynthesis [25]. This process is essential for correct folding, processing, and integration of the protein into the cell membrane, ensuring it reaches the cell surface where it can perform its functions in molecular interactions and signal transduction [25,26,27].

A substantial portion of the FcRL3 protein, spanning amino acids 18–573, forms its extracellular domain [25]. This region contains six C2 domains, structurally similar to Igs. Each domain consists of roughly 78–88 amino acids and is organised in a modular arrangement [25]. According to the UniProt database, the approximate amino acid ranges for the individual domains are [25]

- Domain I (21–98);
- Domain II (99–182);
- Domain III (192–270);
- Domain IV (284–369);
- Domain V (383–470);
- Domain VI (476–563).

The arrangement of Ig-like domains within the extracellular structure is characteristic of many immune receptors and enables versatile ligand interactions. These domains may function independently or cooperatively in recognising specific molecular structures [28].

The transmembrane segment, linking the extracellular and cytoplasmic regions, spans amino acids 574–594 [25]. Its predicted structure is an α-helix composed mainly of hydrophobic residues. This hydrophobic character favours embedding in the lipid bilayer, where it serves as an anchoring element stabilising the position of FcRL3 on the cell surface. Such helical motifs are commonly found in membrane-spanning regions because they allow hydrogen bonds to form even within a non-polar environment.

The terminal portion of FcRL3, encompassing residues 595–734, forms the intracellular domain [25]. This region contains four characteristic ITIMs, each with a defined amino acid sequence, which play a key role in regulating immune signal transduction. According to UniProt data, the ITIMs identified in human FcRL3 are [25].

- ITIM I (648–653, sequence: LTYSSL);
- ITIM II (660–665, SYNSIV);
- ITIM III (690–695, LEYSSL);
- ITIM IV (720–725, LHYQSV).

Additionally, UniProt analysis suggests the presence of disordered regions within the cytoplasmic tail, particularly between residues 603–655 and 695–734 [25]. The concentration of ITIMs in this part of the protein highlights its likely importance as a negative regulator of immune activity, acting through the recruitment of phosphatases following tyrosine phosphorylation [24]. The disordered nature of the terminal fragment may also enhance the accessibility of these motifs for interaction with intracellular signalling proteins. To further explore the structural architecture of FcRL3, it is useful to consider the secondary structure elements within its domains. The extracellular region, which contains the Ig-like domains, is characterised by the presence of a well-conserved structural motif known as the Ig fold [25]. This motif consists of two anti-parallel β-sheets arranged into a β-sandwich. The stability of this fold is often reinforced by a disulphide bridge linking specific cysteine residues within the domain [29]. Between the individual β-strands lie loops that can vary considerably in sequence between different Ig-type domains, and these are often critical for determining ligand-binding specificity.

The predicted disulphide bridges stabilising the extracellular domains of FcRL3, and likely responsible for maintaining their proper structure, are

- Cys44–Cys82 (Domain I);
- Cys120–Cys163 (Domain II);
- Cys211–Cys260 (Domain III);
- Cys309–Cys358 (Domain IV);
- Cys404–Cys451 (Domain V);
- Cys497–Cys544 (Domain VI).

The preservation of these disulphide bonds is crucial for the structural integrity of the extracellular portion of the receptor, ensuring correct alignment of the Ig-type domains and the appropriate exposure of potential binding sites.

Understanding the function of FcRL3 also requires consideration of the spatial arrangement of its six extracellular domains. Although the complete experimental structure of the human FcRL3 molecule has not yet been determined, predictive models have been generated using modern computational tools, particularly the AlphaFold database. The deep learning algorithms employed by AlphaFold enable high-accuracy predictions of protein structure based on amino acid sequence. While these remain computational models, they nonetheless provide valuable insights into the likely spatial organisation of domains. By analogy with other proteins containing multiple Ig-like domains, it is assumed that the FcRL3 domains are arranged either linearly or in a gentle arc above the cell surface [30]. Interactions between neighbouring domains, mediated by specific contact surfaces, may confer a compact extracellular structure. The spatial arrangement and potential flexibility between domains are likely to be critical for ligand recognition and for either triggering or suppressing signalling responses. The transmembrane domain, spanning residues 574–594, is predicted to adopt an α-helical structure composed mainly of hydrophobic residues [30].

This segment is embedded in the lipid bilayer and acts as an anchor, attaching the extracellular domains to the cell surface while positioning the cytoplasmic region inside the cell. The single-pass nature of this transmembrane domain determines the orientation of FcRL3 in the membrane: the portion responsible for ligand binding faces outward, whereas the motifs responsible for signal transduction are accessible within the cytoplasm [30]. This spatial arrangement is essential for the proper reception of signals from the environment and their transmission into the cell to elicit a response.

The cytoplasmic domain of FcRL3, located at the carboxyl terminus of the protein (residues 595–734), contains four ITIMs situated in regions predicted to be partially disordered. The tyrosine residues in these motifs—Y650, Y662, Y692, and Y722—are known to undergo phosphorylation [30]. Once phosphorylated, the ITIMs act as docking sites for intracellular signalling proteins with SH2 domains. These include phosphatases such as SHP-1, SHP-2, and INPP5D, as well as kinases such as Syk and Zap-70 [30]. Phosphorylation of the ITIMs within the cytoplasmic tail of FcRL3 represents the principal mechanism through which this receptor modulates intracellular signalling pathways [24]. Depending on the context and binding partners involved, this can result in either activation or inhibition of cellular responses. Furthermore, the presence of an ITAM-like motif in the cytoplasmic region has been suggested, which may contribute to the dual nature of FcRL3 signalling. Interplay between the inhibitory ITIMs and the potentially activating ITAM-like motif enables fine-tuned regulation of immune cell responses, maintaining the balance between activation and tolerance.

Given the presence of six Ig-like domains in the extracellular portion of FcRL3, it is highly probable that one or more of these domains participate in ligand binding [30]. One known ligand of FcRL3 is secretory IgA (sIgA) [18], with evidence suggesting that specific loops or surface residues within the Ig-like domains form the binding site for IgA [18]. In addition, FcRL3 has been shown to interact with the sperm ligand IZUMO1 during fertilisation, most likely through a defined region of its extracellular domain that recognises IZUMO1 [31]. The capacity of FcRL3 to bind different ligands implies that distinct Ig-like domains, or specific regions within them, mediate these interactions, underscoring the functional versatility of its multi-domain structure. The strong association of *FCRL3* with autoimmune diseases further suggests that genetic polymorphisms within the extracellular domain may modify its ability to bind ligands or interact with other components of the immune system, thereby increasing disease susceptibility. Structural changes arising from such variants may impair the normal function of FcRL3 and contribute to the immune dysregulation characteristic of autoimmunity.

**Figure 1:**
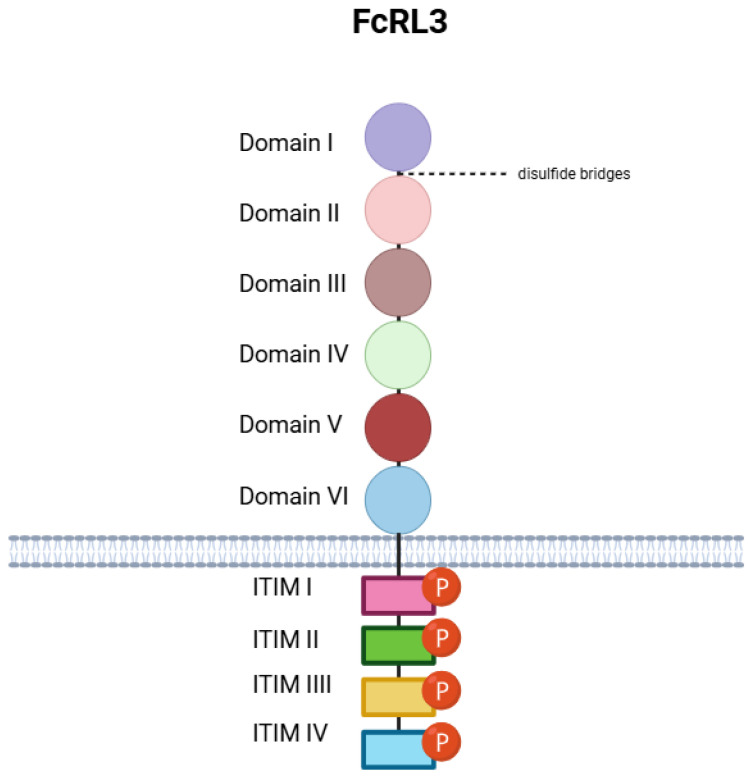
FcRL3 structure. In the extracellular subspace, FcRL3 consists of six domains connected by disulfide bridges. The C-terminal region of FcRL3 extends into the cytoplasm, where there are four ITIMs along with phosphorylated tyrosine residues. Created in BioRender. Plewa, P. (2025) https://BioRender.com/6exm8fz.

To better visualise the spatial organisation of FcRL3, imagine the protein embedded in the cell membrane. The membrane can be represented as a horizontal line, with the extracellular space above and the cytoplasm below. The N-terminal region of FcRL3 projects into the extracellular space, where six Ig-like domains are arranged in a roughly linear sequence, with slight angling between them, resembling a gently curved row of beads [25]. Each domain is labelled sequentially from the N-terminus as “Ig-like domain 1” through “Ig-like domain 6” [25], and within each spherical domain, small connecting lines represent stabilising disulphide bridges. The transmembrane region is depicted as a short, α-helical cylinder embedded in the membrane, labelled “transmembrane helix” [25]. The C-terminal region of FcRL3 extends into the cytoplasm, where four ITIMs are illustrated as short linear segments labelled “ITIM 1” through “ITIM 4,” arranged sequentially from the proximal portion to the C-terminus [25]. Adjacent to each ITIM label is a small circle marked with a “P,” denoting phosphorylated tyrosine residues (Y650, Y662, Y692, Y722). Finally, the predicted disordered regions of the cytoplasmic tail are depicted as flexible or loosely drawn segments, connecting the transmembrane helix to the first ITIM and linking successive ITIMs through to the C-terminus (Figure 1).

In summary, human FcRL3 is a type I transmembrane protein with a distinctive spatial organisation. Its extracellular region comprises six Ig-like domains, likely arranged in a linear fashion, which are responsible for ligand binding. A single transmembrane helix anchors the protein in the cell membrane, while the cytoplasmic tail contains four ITIMs located in partially disordered regions. These structural features are central to the diverse roles of FcRL3 in regulating immune responses, including inhibition of B-cell receptor signalling, as well as its involvement in other biological processes, such as sperm–egg interactions during fertilisation [31,32]. Understanding the structure of FcRL3, particularly in the context of genetic variants, is also key to elucidating the pathogenesis of autoimmune diseases. A detailed knowledge of the molecular architecture of immune receptors such as FcRL3 is crucial both for advancing our understanding of immune system function and for guiding the development of targeted therapeutic strategies for autoimmune disease.

## 4. Molecular Mechanisms of FcRL3 Action in the Pathogenesis of RA: Involvement in Autoantibody Production and Antigen Presentation

**Figure 2:**
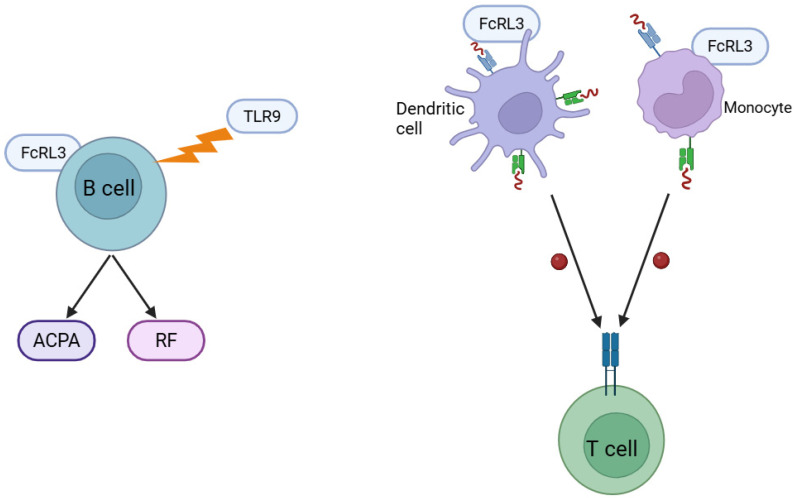
The impact of the FcRL3 receptor on B and T lymphocyte functions: FcRL3, a member of the FcR-like receptor family that modulates immune responses, plays a significant role in regulating the activation, differentiation, and tolerance of both B cells and subsets of T cells (including Tregs). Created in BioRender. Plewa, P. (2025) https://BioRender.com/8u1w3ta.

FcRL3 plays an important role in autoantibody production and antigen presentation, both of which are key stages in the pathogenesis of RA. Expression of FcRL3 on the surface of activated B cells enhances their sensitivity to signals from Toll-like receptors (particularly TLR9), which in turn intensifies the production of autoantibodies, including ACPA and RF—hallmarks of RA [24,33]. A study by Okada et al. [34] identified more than 100 non-*HLA* gene loci critical in RA pathophysiology, and more than half of patients with RA test positive for ACPA [13,35,36]. Increased autoantibody production contributes directly to chronic inflammation and joint destruction by forming immune complexes that activate the complement system and effector cells in the synovial membrane [13]. In addition, FcRL3 may affect antigen presentation by antigen-presenting cells, including dendritic cells and monocytes. Although data remain limited, it has been suggested that this receptor modulates the efficiency of autoantigen presentation to T cells, thereby exacerbating autoimmune processes [23,37]. In this way, FcRL3 may play a dual role: enhancing autoantibody production by B cells while also modulating T-cell responses to presented autoantigens, thus reinforcing the immune mechanisms driving the chronic synovial inflammation characteristic of RA (Figure 2) [21]. The precise molecular mechanisms by which FcRL3 regulates antigen presentation remain to be clarified, but current evidence highlights its significant potential as a regulator of autoimmune processes in RA.

Current research indicates that FcRL3 has considerable potential both as a prognostic biomarker and as a therapeutic target in RA. From a prognostic perspective, FcRL3 expression may serve as a valuable marker for identifying patients at risk of aggressive disease progression and rapid joint destruction. Elevated FcRL3 expression is frequently associated with poorer outcomes, including faster development of radiological changes, resistance to standard therapies, and higher titres of autoantibodies (ACPA, RF). These associations highlight the clinical value of FcRL3 in predicting the course of RA. This may also translate into an increased risk of cardiovascular or thrombotic diseases. This process may occur through Treg modulation, which may increase the risk of cardiovascular disease in RA [23,38,39]. Consequently, assessing FcRL3 expression could become a useful tool for early risk stratification and the personalisation of treatment strategies, helping to limit disease progression from its earliest stages [23,40].

Analyses of FcRL3 expression in the synovial membrane of patients with RA further support its role in local inflammatory processes. Synovial tissue with higher FcRL3 expression is characterised by dense inflammatory infiltrates, dominated by activated B and T cells, and accompanied by heightened production of pro-inflammatory cytokines (including TNF-α, IL-6, and IL-17) as well as accelerated joint destruction [1,14]. Kochi et al. [38] also demonstrated that increased FcRL3 expression correlates with a higher risk of rapid radiological progression, reinforcing its potential as a prognostic marker of aggressive disease. Similarly, Wang et al. [39] reported that FcRL3 expression is particularly elevated in patients with newly diagnosed, active RA, and that receptor expression levels on B cells correlate positively with DAS28 disease activity scores—evidence of its direct involvement in the initiation and persistence of inflammation and autoimmunity in RA [9,21].

Another important aspect of the clinical significance of FcRL3 is its potential influence on therapeutic response. Observations suggest that patients with high expression of this receptor may respond less effectively to standard immunosuppressive therapies, including conventional disease-modifying antirheumatic drugs as well as biological agents such as TNF-α inhibitors or abatacept [37,41]. The underlying mechanism is thought to involve modulation of activating and inhibitory signals, whereby FcRL3 promotes the survival and activation of autoreactive B cells while reducing the immunoregulatory efficacy of Tregs, thus contributing to resistance against standard treatment regimens [21,23,24]. Consequently, assessing FcRL3 expression could help identify patients who may require more intensive or alternative therapies early in the disease course. Genome-wide association studies (GWAS) have also advanced our understanding of autoimmune diseases, including RA. Genes within the HLA-DRB region are the most strongly correlated with RA risk [34,42,43,44]. A study by Okada et al. [34] identified more than 100 non-*HLA* gene loci critical to RA pathophysiology, and more than half of patients with RA test positive for ACPA [13,35,36]. Importantly, these antibodies may be present years before the onset of RA symptoms. ACPA positivity has been associated with a more severe disease course, frequently accompanied by visceral involvement, faster progression of joint deformities, and worsening of comorbidities. Moreover, the presence of ACPAs is linked to increased mortality risk. There is a statistically significant association between ACPA status and the *FCRL3* SNP rs2317230, with an odds ratio (OR) of 3.1 (95% confidence interval: 1.14–8.48) [45,46,47].

## 5. Association of FcRL3 Polymorphism with RA in Different Populations

Genetic studies have demonstrated a significant association between an SNP in the promoter region of the *FCRL3* gene, designated −169C/T (rs7528684), and an increased risk of RA. This variant was first identified as a susceptibility factor in the Japanese population, where the presence of the T allele correlated with elevated *FCRL3* expression and a markedly higher risk of RA, particularly in ACPA- and RF-seropositive individuals [48,49]. Subsequent studies in other ethnic groups yielded partly divergent results. While the association was consistently confirmed in Asian populations (Chinese, Korean), findings from European cohorts were less conclusive, suggesting possible ethnic variability or the influence of additional environmental factors on disease development [50,51,52]. Mechanistically, the −169C/T variant appears to influence RA risk through modulation of *FCRL3* promoter activity, altering NF-κB transcription factor binding. The T allele increases *FCRL3* transcription, which may promote immune dysregulation and susceptibility to autoimmunity [48]. Meta-analyses of available studies indicate that rs7528684 is a moderate but significant genetic risk factor for RA, especially within certain population groups and specific seropositive disease subtypes [53]. Cohort studies across different ethnicities reinforce this conclusion, although with important variation in effect size. In Japanese and Korean cohorts, a strong association was observed between the T allele and RA risk, particularly among ACPA- and RF-positive patients, supporting a pronounced genetic contribution in these populations [48]. Chinese studies also confirmed a moderate but significant link, although weaker than in Japanese cohorts [54]. By contrast, results from European populations have been inconsistent. Studies from the Netherlands and Norway reported a moderate association between the homozygous CC genotype and increased RA risk, a pattern differing from Asian findings [50,51]. However, other analyses—such as those in British cohorts—found no clear evidence of a significant association between this polymorphism and RA susceptibility [55]. Overall, meta-analyses spanning diverse cohorts suggest that rs7528684 exerts a moderate but significant influence on RA risk, while also underscoring the importance of ethnic and genetic variability. These findings point to the need for future studies that consider gene–environment interactions to fully clarify the role of *FCRL3* polymorphisms in RA pathogenesis [53,56,57,58].

## 6. *FCRL3* as a Negative Regulator of Treg Function and Receptor for sIgA

**Figure 3:**
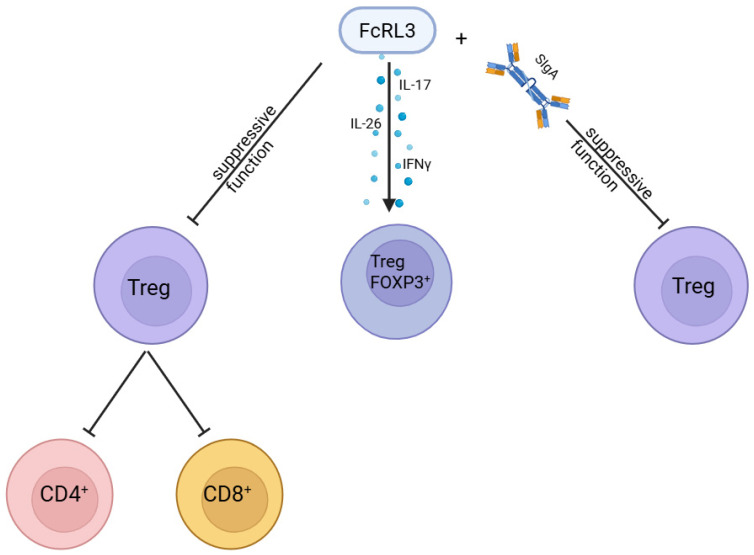
Effect of FcRL3 on Treg cells: The FcRL3 receptor modulates their suppressive functions and phenotypic stability. Its expression has been associated with immune dysregulation. Created in BioRender. Plewa, P. (2025) https://BioRender.com/oo259sz.

Beyond the correlation between *FCRL3* polymorphisms and RA, it is important to examine the mechanisms of FcRL3 action in more detail. A key finding was the demonstration that stimulation of FcRL3 inhibits the suppressive function of Treg cells. This was confirmed in vitro by Agarwal et al. [18] using an FcRL3-specific antibody. In their experiment, sorted CD4^+^CD25CD127^−^ Tregs were labelled with violet dye to distinguish them from responder cells in culture. These Tregs were then incubated with CD3, FcRL3, or TLR2 ligands before being co-cultured with autologous responder cells stimulated with CD3/CD28 beads and labelled with CFSE. The results showed that FcRL3 stimulation blocked the ability of Tregs to suppress the proliferation of CD4^+^ and CD8^+^ responder cells, an effect comparable to stimulation with TLR2 ligands (used as a positive control) (Figure 3). CD3 stimulation alone had no effect, while co-stimulation with CD3 and FcRL3 produced results similar to FcRL3 stimulation on its own. Comparable findings were obtained when sorted CD4^+^ responder cells were tested. Because in vitro experiments alone could not fully verify this observation, the study was extended to lymphoid tissue adjacent to the mucosa. Tonsillar tissue analysis revealed that 40.1 ± 14.5% (mean ± SD, n = 7) of Tregs expressed FcRL3. These results confirm that FcRL3 functions as an inhibitor of Treg suppressive activity.

It has been documented that Treg cells can transform into Th17 cells, and in the context of FcRL3 this may explain the reduced suppressive activity of Tregs following receptor stimulation [59]. To investigate this, cytokines typically produced by Th17 cells—IL-17, IL-22, IL-26, and interferon-γ (IFN-γ)—were assessed [60]. FcRL3-stimulated Treg cells and responder cells were co-cultured under the same conditions as in the Treg suppression assay. After 15 and 24 h, an increased percentage of Treg cells expressing IL-26 and IL-17 was observed in response to FcRL3 stimulation. IL-22 expression was not induced, while IFN-γ levels rose only moderately. Both FcRL3 stimulation alone and co-stimulation with CD3 promoted a marked increase in IL-17 expression and enhanced IL-26 secretion. By contrast, CD3 or TLR2 stimulation alone had no comparable effect. Interestingly, FcRL3 stimulation did not reduce FOXP3 expression. One day after stimulation, 54% of Treg cells were double-positive for FOXP3 and RORγt [18], indicating that while the cells retained their Treg identity, they simultaneously acquired a Th17-like phenotype. A substantial proportion of FOXP3-positive cells contained IL-17 protein. Taken together, these findings suggest that FcRL3 stimulation induces production of pro-inflammatory cytokines IL-17, IL-26, and IFN-γ by driving the development of FOXP3-positive Treg cells that co-express RORγt [18] (Figure 3).

Wilson et al. [60] demonstrated that FcRL3 does not bind IgG, IgA, or IgM, a finding later confirmed by Agarwal et al. [18]. In addition to clarifying the role of FcRL3 as a negative regulator of Tregs, Agarwal et al. [18] also showed that sIgA, unlike plasma IgA, binds to FcRL3 and in doing so weakens the suppressive function of regulatory T cells (Figure 3) [18]. This interaction is characterised by moderate affinity (0.45 ± 0.32 μM) and follows a simple 1:1 kinetic model, with both association and dissociation occurring slowly. Protein fraction analysis revealed that only sIgA containing both the J chain and the secretory component is capable of binding FcRL3, whereas dimeric and monomeric forms of IgA lack this ability [18]. These findings suggest that sIgA may represent a physiological ligand for FcRL3, modulating Treg activity and thereby influencing immune regulation. The binding is proportional to FcRL3 expression levels and can be blocked using an anti-FcRL3 antibody, confirming the specificity of the interaction.

## 7. FcRL3 Expression in the Immune System: Regulation Under Physiological and Inflammatory Conditions

The regulation of FcRL3 expression is a dynamic process that depends on both the activation state of the immune system and external stimuli. Under physiological conditions, FcRL3 expression is relatively low, found mainly on resting B and T cells, where it functions as a regulator of signalling in response to subtle receptor inputs such as B-cell receptor (BCR) or TLR stimulation [23,24]. By contrast, pro-inflammatory factors such as IL-2, IL-15, and IFN-γ markedly increase FcRL3 expression on activated B and T cells, indicating a role in modulating the amplified immune responses characteristic of chronic inflammation [21,41]. Elevated FcRL3 expression has also been observed in inflamed tissues of patients with autoimmune diseases, including RA and systemic lupus erythematosus, supporting its direct involvement in disease pathogenesis [33,41]. At the transcriptional level, regulation of FcRL3 is strongly influenced by genetic variation in its promoter region (rs7528684, −169C/T). This polymorphism may alter NF-κB binding and thereby enhance *FCRL3* transcription in response to inflammatory stimuli [61]. Collectively, these mechanisms suggest that FcRL3 is a key regulator of inflammatory immune responses—helping to maintain immune homeostasis under normal conditions but contributing to autoimmunity when dysregulated.

An important mechanism of FcRL3 action in the pathogenesis of RA is the modulation of activation signalling by BCRs and T-cell receptors (TCRs). A well-established feature of natural Treg (nTreg) cells is their weak response to TCR stimulation in culture, a response that can be enhanced by the addition of exogenous IL-2 [61,62]. It is important to note the distinction between Treg function in vitro and in vivo; nonetheless, in this context the crucial role of IL-2 has also been demonstrated in vivo [63]. Nagata et al. [41] showed that IL-2 promoted in vitro proliferation of FcRL3− nTreg cells, whereas FcRL3+ nTreg cells did not proliferate under the same conditions. This finding suggests that IL-2 stimulates anti-apoptotic processes in nTregs in a manner closely linked to FcRL3 expression. Supporting this, mouse nTregs cultured in vitro with IL-2 temporarily lost their suppressive function but spontaneously regained it following IL-2 withdrawal [62]. No correlation between TCR stimulation and FcRL3 expression was observed in the CD8+ cell fraction. At the molecular level, FcRL3 is characterised by the presence of both inhibitory (ITIM) and activating (ITAM-like) motifs in its cytoplasmic domain. This dual structure allows it to function as a bidirectional regulator of lymphocyte activation, depending on the immunological context [23,24]. In B cells, FcRL3 expression can amplify signalling through Toll-like receptors, particularly TLR9, thereby promoting activation of autoreactive cells and increasing the production of autoantibodies such as ACPAs, characteristic of RA [24,33]. At the same time, FcRL3 may inhibit plasma cell differentiation, reflecting its complex role in modulating the humoral immune response and sustaining RA-associated inflammation [24]. In T cells, particularly the regulatory subpopulation, elevated FcRL3 expression is linked to reduced suppressive activity. This occurs, in part, through increased expression of the PD-1 checkpoint receptor and reduced IL-2 production, both of which undermine immune tolerance and contribute to chronic autoimmunity [21,41]. Thus, FcRL3 plays a dual role in RA pathogenesis: enhancing the activation of autoreactive lymphocytes while simultaneously weakening the regulatory mechanisms of the immune system. Together, these processes support the persistence of chronic inflammation characteristic of RA [23,37].

## 8. The Role of *FCRL3* in Regulating B-Cell Responses to TLR9 Stimulation by CpG Sequences

**Figure 4:**
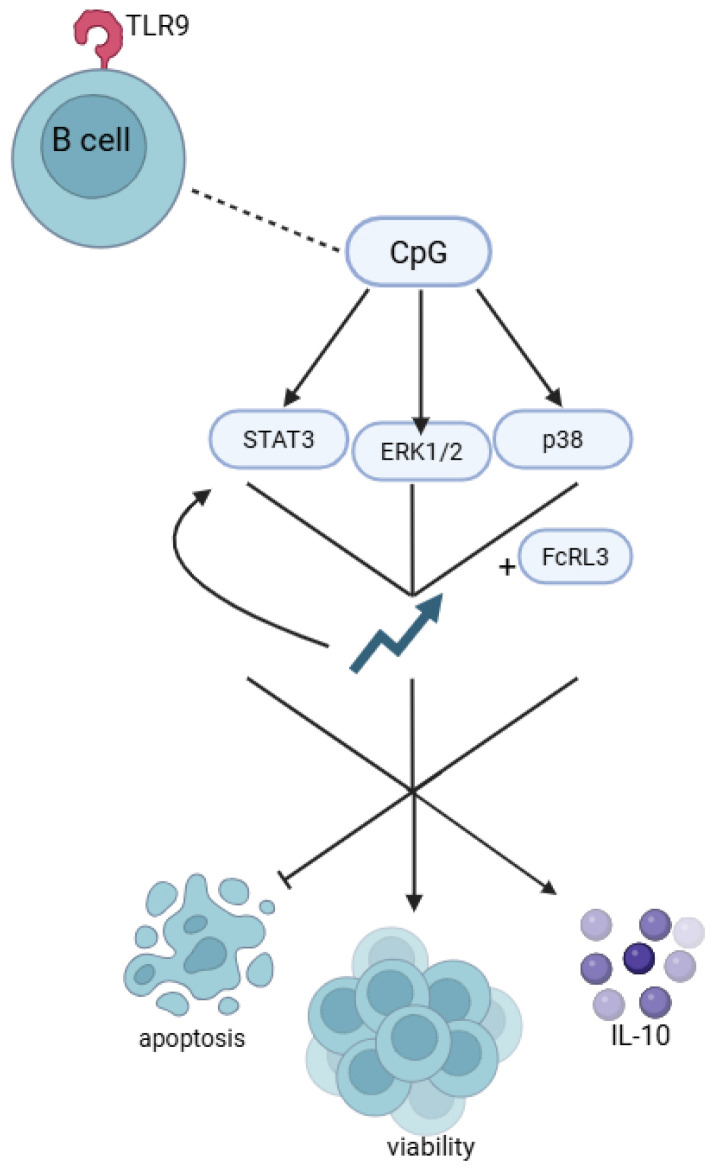
The Role of FcRL3 in regulating B cell response to TLR9 stimulation by CpG sequences: The FcRL3 receptor, expressed on B cells, modulates their response to TLR9-dependent signaling induced by CpG DNA sequences. Its presence influences cytokine secretion, apoptosis, and B cell viability, which may be important for their function in the immune response and disease pathogenesis. Created in BioRender. Plewa, P. (2025) https://BioRender.com/1rgy8hl.

Liu et al. [64] demonstrated that TLR9 activation in B cells by CpG sequences promotes *FCRL3* expression. CpG sites are DNA regions in which a cytosine nucleotide is immediately followed by a guanine nucleotide in the 5′ → 3′ sequence. These sites occur at high frequency within genomic regions known as CpG islands. According to the study, CpG sequences significantly activated the STAT3, ERK1/2, and p38 pathways. Increased *FCRL3* expression further enhanced the activation of these signalling pathways, while silencing with *FCRL3* siRNA markedly reduced their activation in response to CpG [64]. Functionally, CpG stimulation inhibited B-cell apoptosis, increased cell viability, and promoted both antibody production and IL-10 secretion. Enhanced expression of FcRL3 augmented the pro-survival and IL-10-inducing effects of CpG, while at the same time suppressing the CpG-driven increase in antibody production (Figure 4). Conversely, *FCRL3* siRNA blocked most of the regulatory effects of CpG stimulation, but under these conditions antibody production by B cells was increased [64]. The use of ERK1/2, STAT3, and p38 pathway inhibitors abolished the effects of CpG stimulation namely, the promotion of cell viability, antibody production, and IL-10 secretion. These findings indicate that FcRL3, via its cytoplasmic ITIMs, modulates B-cell responses to TLR9 stimulation, particularly antibody and IL-10 secretion [64]. Taken together, the data suggest that TLR9 stimulation by CpG sequences enhances B-cell proliferation, reduces apoptosis, and induces both antibody production and IL-10 secretion through the ERK1/2, STAT3, and p38 pathways, with FcRL3 acting as a key regulatory element in this process [64].

## 9. *FCRL3* Polymorphisms and Anti-ApoA-1 IgG Antibody Response

Autoimmune diseases account for a substantial proportion of chronic conditions and markedly impair quality of life, affecting up to 10% of the population [65,66,67]. Chronic inflammation arising from an immune response not only causes local discomfort but also leads to systemic complications. Activation of B cells—a hallmark of humoral autoimmunity—can exacerbate health deterioration even when symptoms are mild [68,69,70]. Less common autoimmune disorders may result from monogenic mutations, whereas common diseases such as RA reflect complex interactions between environmental and multigenic risk factors that disrupt immune tolerance. Thanks to GWAS, it has become possible to link even subtle dysregulation of the immune system to common genetic variants predisposing to clinically significant autoimmunity [71,72], with autoantibody production being one of the most detectable outcomes [73,74,75]. Although the risk conferred by individual variants is relatively modest, modern GWAS methods, which efficiently screen for such variants, allow objective assessment of the biological pathways driving autoimmunity [65,66,67,76]. A key finding from the study by Antiochos et al. [77] was the demonstration of a link between *FCRL3* polymorphisms and anti-apoA-1 IgG antibody levels. The SNP rs6427397, an intergenic variant, was shown to influence *FCRL3* expression in blood [76]. The correlation with anti-apoA-1 IgG levels aligns with earlier observations that more than 60% of loci associated with autoimmunity in GWAS are shared across multiple autoimmune diseases [66,67,78,79]. According to Plagnol et al. [73], an *FCRL3* locus is associated with antibodies to the insulinoma-2-related antigen, suggesting that *FCRL3* plays an important role in the interactions between T cells and antigen-presenting cells that ultimately drive the generation of antibody-producing plasma cells. However, Antiochos et al. [77] did not examine relationships between FcRL3 and other autoantibodies such as antinuclear antibodies, heat-shock protein antibodies, antiphospholipid antibodies, or oxidised LDL antibodies—data that could have further tested this hypothesis. Their study nevertheless found that the T allele of rs6427397 exerted a moderate effect on antibody levels [OR, 1.27 (95% CI, 1.15–1.40)], explaining 0.67% of the variability in anti-apoA-1 IgG titres [77]. Such modest effect sizes are in line with the clinical impact of numerous genetic variants in autoimmunity research, where risk alleles typically have odds ratios below 1.2 [71,72,74]. Although genomic risk variants may not be directly useful for assessing individual disease risk, they provide crucial aetiological information on the regulatory regions involved in disease pathogenesis. Genetic markers of this kind can be used to investigate responses to targeted immune-modulating therapies, to explain disease variability, and to inform the rational development of new drugs. The main SNP investigated in this study lies in a non-coding, intergenic region at the *FCRL3* locus. Prior evidence has shown that around 60% of non-coding variants function as enhancers—regulatory sequences that control gene activity in specific immune cell types—and that roughly 90% of DNA variants most strongly linked to autoimmune disease are non-coding [65]. Current knowledge therefore suggests that intergenic regions harbour numerous regulatory elements that shape gene expression in a cell type–specific manner [65,66]. Supporting this, Kochi et al. [38] reported that non-coding SNPs in the *FCRL3* promoter region influence gene expression, leading to increased autoantibody production in genetically susceptible individuals.

## 10. The *FCRL* Gene Family in the Pathophysiology of Diseases

The *FCRL* gene family comprises six members: *FCRL1*, *FCRL2*, *FCRL3*, *FCRL4*, *FCRL5*, and *FCRL6*. *FCRL6* is mainly expressed by T cells and NK cells [80], whereas *FCRL1–5* are expressed predominantly in B cells. The expression of *FCRL* genes has been investigated in the context of malignant tumours such as mantle cell lymphoma and multiple myeloma [79,80].

FcRL proteins exhibit marked functional diversity, owing both to the structural variation in their Ig-like domains and to the distinct signalling motifs present in their cytoplasmic regions. FcRL1, FcRL2, and FcRL4 are expressed mainly on B cells, where they modulate BCR signalling and thereby regulate B-cell activation, proliferation, and differentiation [23,37]. FcRL5, by contrast, is involved primarily in the regulation of the later stages of B-cell maturation and influences plasma cell differentiation [80]. FcRL6 is notable for its expression on T cells and NK cells, where it contributes to the modulation of cytotoxicity and proliferative activity [80]. Among this family, FcRL3 is of particular interest because of its broad expression profile, spanning B cells, T cells, and NK cells. Unlike other family members, FcRL3 contains both activating (ITAM-like) and inhibitory (ITIM) motifs, enabling it to act as a bidirectional modulator of immune responses [24]. Functionally, FcRL3 exerts significant influence over B-cell activity by modulating responses to TLR9 signalling and over T cells by regulating PD-1 expression. These properties are thought to be important for the breakdown of immune tolerance in autoimmune diseases such as RA [21,24].

## 11. Challenges and Prospects for the Development of New FcRL3-Targeted Drugs for the Treatment of RA

The development of new therapeutic strategies targeting FcRL3 is an important area of translational research in RA but involves a number of significant scientific and clinical challenges. A central issue is the complex and still not fully understood biology of FcRL3. This receptor contains both activating (ITAM-like) and inhibitory (ITIM) motifs, enabling simultaneous stimulation and inhibition of various signalling pathways in immune cells [23,37]. Effective FcRL3-targeted treatment therefore requires precise determination of the timing and immunological context in which the receptor’s function should be inhibited or enhanced. Inadequate modulation could lead to unintended consequences, such as excessive immunosuppression, increased risk of infection, or even exacerbation of autoimmune processes rather than their suppression [1,40]. Another key challenge is the heterogeneity of the RA patient population and the resulting need for personalised treatment. Genetic polymorphisms—such as the rs7528684 (−169C/T) variant of the *FCRL3* gene—inter-individual differences in receptor expression, and diversity in immunological disease phenotypes may all contribute to variability in responses to FcRL3-targeted therapy [24,38]. For this reason, continued research into biomarkers is crucial to identify patients most likely to benefit. In this context, a major goal for future studies is the development of molecular biomarker panels to improve patient selection and allow precise monitoring of therapeutic efficacy [1,24,38]. A further significant challenge lies in the technical demands of developing biological drugs and small-molecule inhibitors specific to FcRL3. The generation of monoclonal antibodies with high specificity and appropriate pharmacokinetic and pharmacodynamic properties is time-consuming and costly [39]. Preclinical studies to date indicate potential efficacy of anti-FcRL3 antibodies, but additional clinical trials are needed to confirm their safety and effectiveness in patients with RA [21,41]. Moreover, optimal dosing regimens must be defined with care to avoid adverse effects, particularly those arising from chronic modulation of the immune system [39,40].

## 12. Therapeutic Potential Targeting FcRL3 and Previous Attempts at Modulation Using Anti-FcRL3 Antibodies and Signalling Pathway Inhibitors

**Figure 5:**
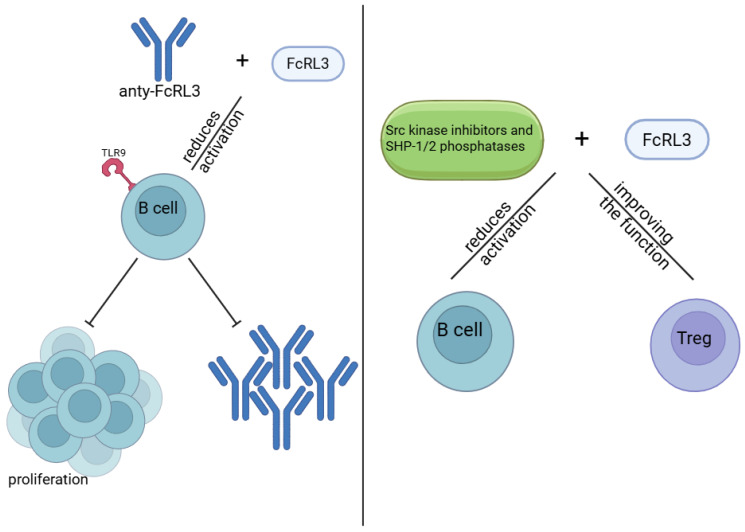
Therapeutic Potential of Targeting FcRL3 with Antibodies and Inhibitors: Modulation of FcRL3 receptor function represents a promising therapeutic strategy for the treatment of RA. Antibodies and inhibitors directed against FcRL3 may influence the regulation of B and T lymphocyte activity, restoring immune balance and inhibiting pathological inflammatory processes. Created in BioRender. Plewa, P. (2025) https://BioRender.com/wk11zag.

Given the documented role of FcRL3 in regulating immune responses and its involvement in the pathogenesis of autoimmune diseases such as RA, this receptor represents an attractive target for new therapeutic strategies. Previous attempts to modulate FcRL3 have mainly involved experimental approaches using anti-FcRL3 monoclonal antibodies, as well as targeted interventions in signalling pathways associated with this receptor. Preliminary studies at the cellular level and in animal models have shown that antibodies directed against FcRL3 can effectively modulate the activation of B and T cells, thereby inhibiting autoimmune processes and reducing autoantibody production [23,37]. By specifically engaging the extracellular domain of FcRL3, anti-FcRL3 antibodies have the potential to dampen activation of Toll-like receptors (e.g., TLR9), key mediators of immune responses implicated in autoantibody generation in RA [24]. For example, Li et al. [24] demonstrated that blocking FcRL3 with specific monoclonal antibodies reduces B-cell activation in response to TLR9, lowering both proliferation and antibody production, and thereby suggesting potential clinical utility for controlling excessive humoral responses in RA. In parallel, efforts have focused on pharmacological modulation of signalling pathways activated by FcRL3. Owing to the presence of both activating (ITAM-like) and inhibitory (ITIM) sequences in its cytoplasmic domain, FcRL3 can influence the activity of various protein kinases and phosphatases, including Src-family kinases (e.g., Lyn) and the phosphatases SHP-1 and SHP-2 [21,33]. Experimental inhibitors of these enzymes may therefore modulate FcRL3 activity, leading to reduced inflammation and attenuation of signals that promote autoimmunity. Preclinical studies indicate that inhibition of Src kinases and SHP-1/2 can reduce B-cell activation and improve regulatory T-cell (Treg) function, changes that could be beneficial in RA (Figure 5) [21,41]. Despite these promising findings, no full-scale clinical trials have yet evaluated the safety and therapeutic efficacy of specific FcRL3 inhibitors in patients with RA. Important questions remain about potential adverse effects associated with perturbing FcRL3 function—particularly given its complex role in immune regulation and the attendant risk of excessive immunosuppression [40]. A further challenge is ensuring sufficient selectivity to avoid unwanted interference with related receptors within the FcRL family.

In summary, attempts to date to modulate FcRL3 using monoclonal antibodies and signalling pathway inhibitors highlight the considerable therapeutic potential of this strategy. Nevertheless, further research is required to establish its clinical utility, safety, and optimal application, which may ultimately represent a breakthrough in the treatment of RA and other autoimmune diseases.

## 13. Perspective for the Future

However, there remain important knowledge gaps requiring further investigation. First, the detailed molecular mechanisms and full functional spectrum of FcRL3 in autoimmune disease are not fully defined. In particular, the precise role of FcRL3 in antigen-presentation processes and its impact on the function of regulatory immune cell subpopulations (e.g., Treg cells, regulatory B cells) remain to be elucidated. In addition, further analysis of ethnic and genetic differences in FcRL3 expression and function is needed to enable better personalisation of potential therapies. There is also a lack of clinical trial data evaluating the efficacy and safety of drugs targeting FcRL3 in patients with RA, which is essential for eventual translation into clinical practice.

At the same time, growing understanding of the molecular mechanisms underpinning FcRL3 involvement in RA pathogenesis supports the concept of using FcRL3 as a potential therapeutic target.

With its ability to modulate both activating and inhibitory immune signals, FcRL3 influences key aspects of the immune response, including autoantibody production by B cells and the function of Tregs. Targeted therapy against FcRL3 could, in principle, reduce the activation of autoreactive B cells and restore normal Treg function, thereby diminishing chronic arthritis and slowing musculoskeletal damage. At present, however, therapeutic strategies directed specifically at FcRL3 remain at the preclinical stage, and further studies are required to evaluate their safety and clinical efficacy. Future research should therefore prioritise several key areas. First, detailed molecular and cellular analyses are needed to define precisely how FcRL3 operates in different immune contexts. Second, large-scale clinical studies are necessary to assess the value of FcRL3 as a prognostic biomarker and to establish the true therapeutic potential of strategies targeting it. Third, there is a need to identify biological parameters that will allow accurate stratification of patients most likely to benefit from FcRL3-targeted therapies, thereby supporting more personalised and optimised treatment for RA.

## 14. Conclusions

**Figure 6:**
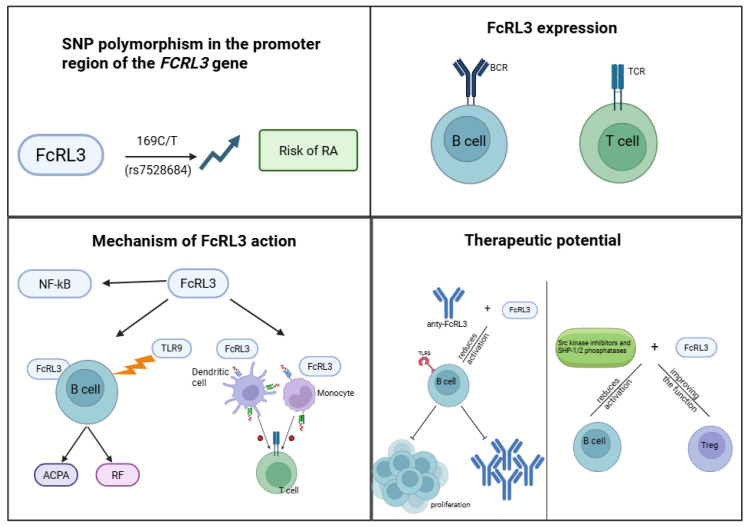
The impact of the FCRL3 gene promoter polymorphism (rs7528684, −169C/T) on the regulation of FCRL3 receptor expression on the surface of T and B cells, and the associated signaling mechanisms and therapeutic potential. The presence of the T allele leads to increased binding of the transcription factor NF-κB to the promoter region, resulting in elevated FCRL3 transcription and increased receptor expression on immune cells. FCRL3 expression on T cells may affect their suppressive function, while on B cells it may modulate immune responses and signaling through immunoglobulin receptors. NF-κB activation, as a key regulator of the inflammatory response, constitutes a central mechanism integrating environmental and genetic signals. The rs7528684 polymorphism may be associated with a predisposition to autoimmune diseases (e.g., RA) through dysregulation of immune tolerance. Understanding this mechanism opens new therapeutic possibilities. Created in BioRender. Plewa, P. (2025) https://BioRender.com/zgsqnxc.

Previous findings clearly indicate that FcRL3 plays an important role in the pathogenesis of RA, affecting multiple aspects of the immune response. Key observations include confirmation of the association between the promoter polymorphism −169C/T (rs7528684) of the *FCRL3* gene and increased disease risk and severity. Expression of FcRL3 on immune cells—particularly B and T cells—has also been shown to correlate with disease activity, autoantibody production (ACPA, RF), and poorer treatment response, indicating the potential of FcRL3 as a prognostic biomarker. In addition, FcRL3 affects immune tolerance through modulation of BCR and TCR signalling, thereby promoting chronic inflammation and increased autoantibody production. Current preclinical studies further indicate that FcRL3 activity can be modulated by specific monoclonal antibodies and signalling pathway inhibitors, offering promising therapeutic prospects (Figure 6).

In conclusion, FcRL3 represents a significant and promising target for research into the pathogenesis and treatment of RA. Despite substantial advances, further work is essential to clarify its biological role, to develop effective targeted therapies, and ultimately to integrate FcRL3 into the routine diagnosis and management of RA.

## Data Availability

No new data were created or analyzed in this study.

## Abbreviations

The following abbreviations are used in this manuscript:

ACPAs	Anti-citrullinated protein antibodies
FCRH	Fc receptor homologue
FcRL3	Fc-like receptor 3
GWAS	Genome-wide association studies
IFN-γ	Interferon-γ
IL	Interleukin
IRTA	Immunoglobulin superfamily receptor translocation associated
ITAM	Immunoreceptor tyrosine-based activation motif
ITIM	Immunoreceptor tyrosine-based inhibitory motif
RA	Rheumatoid arthritis
RF	Rheumatoid factor
SNP	Single-nucleotide polymorphism
TNF-α	Tumour necrosis factor-α
Treg	Regulatory T-cell
